# Restructuring of Pancreatic Islets and Insulin Secretion in a Postnatal Critical Window

**DOI:** 10.1371/journal.pone.0000035

**Published:** 2006-12-20

**Authors:** Cristina Aguayo-Mazzucato, Carmen Sanchez-Soto, Victoria Godinez-Puig, Gabriel Gutiérrez-Ospina, Marcia Hiriart

**Affiliations:** 1 Department of Biophysics, Institute of Cellular Physiology, National Autonomous University of México Mexico City, Mexico; 2 Department of Cell Biology and Physiology, Biomedical Research Institute, National Autonomous University of México Mexico City, Mexico; 3 Faculty of Medicine, National Autonomous University of Mexico Mexico City, Mexico; University of California, Los Angeles, United States of America

## Abstract

Function and structure of adult pancreatic islets are determined by early postnatal development, which in rats corresponds to the first month of life. We analyzed changes in blood glucose and hormones during this stage and their association with morphological and functional changes of alpha and beta cell populations during this period. At day 20 (d20), insulin and glucose plasma levels were two- and six-fold higher, respectively, as compared to d6. Interestingly, this period is characterized by physiological hyperglycemia and hyperinsulinemia, where peripheral insulin resistance and a high plasmatic concentration of glucagon are also observed. These functional changes were paralleled by reorganization of islet structure, cell mass and aggregate size of alpha and beta cells. Cultured beta cells from d20 secreted the same amount of insulin in 15.6 mM than in 5.6 mM glucose (basal conditions), and were characterized by a high basal insulin secretion. However, beta cells from d28 were already glucose sensitive. Understanding and establishing morphophysiological relationships in the developing endocrine pancreas may explain how events in early life are important in determining adult islet physiology and metabolism.

## Introduction

Blood glucose levels in adult mammals are regulated by pancreatic islet hormones, secreted by alpha and beta cells. Early postnatal pancreatic development is important to reach an effective glycemic control in the adult.

A critical window is defined as a period of important structural and functional changes during normal organ development when exposure to certain environmental changes may originate life-time consequences [1].

The pancreatic gland derives from different embryological structures; the head comes from the ventral bud, whereas the body and tail derive from the dorsal bud of the caudal foregut [2]. Fetal and neonatal beta cells show low sensitivity to glucose and scarce, unimodal, insulin secretion as compared with adult beta cells [3]–[5], this condition reflects functional immaturity. During the first month of life, rats are exposed to critical changes that start with birth, followed by the lactation period and finally weaning around d21. A decrease in beta cell proliferation and an increase in apoptotic events that peak between postnatal d13 and d17 have been observed in rodents [6]. It is not fully understood when a mature secretory response is acquired and if this scenario coincides with a critical developmental window that could be associated with major morphophysiological reorganization of the islet.

Diets with high carbohydrate content during lactation can cause sustained hyperinsulinemia and obesity in adult rats [7]–[9]. Also alterations in islet size, number and composition have been observed in response to nutritional changes [10]. These changes may derive in a higher risk for type 2 diabetes development [8], [9].

The aim of this study was to analyze postnatal morphological and functional maturation of pancreatic islets during the first month of life in the rat, because lactation and weaning periods constitute a critical window in metabolic development. In fact, we observed major differences and heterochronic development of islets throughout the gland that could reflect their dual embryological origin. Functionally these changes were paralleled to important metabolic changes, such as hyperinsulinemic hyperglycemia around weaning that stabilizes around d28, where a structure and function similar to adults was observed.

## Results

### Changes in glucose, insulin and glucagon levels in plasma during the first month of life

**Figure 1:**
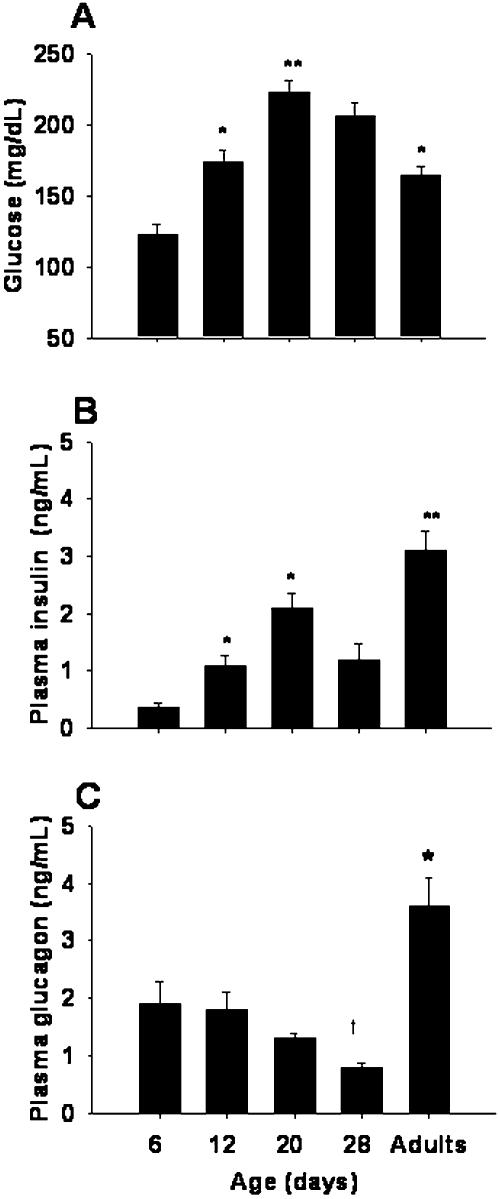
Plasmatic glucose and hormone levels at different ages. **(A)** Glucose concentration (n  =  12 animals/point); **(B)** plasma levels of insulin (n  =  12), and **(C)** plasma levels of glucagon (n  =  6). Symbols denote statistically significant differences, (^*^) p<0.05 and (**) p<0.0001 with respect to previous age, (†) p<0.05 with respect to d6.

Body and pancreatic weight gain was recorded during the first month (Table 1). Plasma glucose levels consistently increased during the first three weeks of life, reaching plasma values of 223 mg/dL on d20 (Fig. 1A). When expressed per gram of body weight, to compensate for growth changes during this period (Table 1), blood glucose concentration was similar from d6 to d20; followed by an 8-fold reduction in adulthood.

**Table 1:**
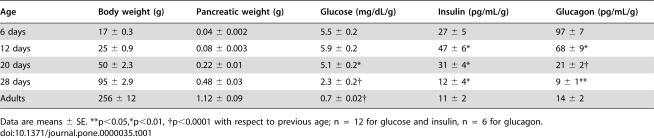
Body and plasmatic parameters measured during the first month of life; plasma glucose, insulin and glucagon expressed per gram of body weight.

Age	Body weight (g)	Pancreatic weight (g)	Glucose (mg/dL/g)	Insulin (pg/mL/g)	Glucagon (pg/mL/g)
6 days	17 ± 0.3	0.04 ± 0.002	5.5 ± 0.2	27 ± 5	97 ± 7
12 days	25 ± 0.9	0.08 ± 0.003	5.9 ± 0.2	47 ± 6*	68 ± 9*
20 days	50 ± 2.3	0.22 ± 0.01	5.1 ± 0.2*	31 ± 4*	21 ± 2†
28 days	95 ± 2.9	0.48 ± 0.03	2.3 ± 0.2†	12 ± 4*	9 ± 1**
Adults	256 ± 12	1.12 ± 0.09	0.7 ± 0.02†	11 ± 2	14 ± 2

Data are means ± SE. **p<0.05,*p<0.01, †p<0.0001 with respect to previous age; n  =  12 for glucose and insulin, n  =  6 for glucagon.

Plasma insulin levels peaked at d20 reaching values 6-fold higher than those observed at d6 (Fig. 1B). When expressed per gram of body weight, plasma insulin levels increased between d6 and d12 and then decreased steadily from d12 to d28, when adult values were reached. Plasma glucagon levels decreased progressively from d6 until d28, where they reached values significantly lower than at d6 (Fig. 1C).

### Structural reorganization of the developing pancreas

We explored structural changes of beta and alpha cells that might be associated with changes in circulating hormone levels. In addition to alpha and beta cells, adult pancreatic islets also contain delta and PP cells that secrete somatostatin and pancreatic polypeptide, respectively. These two populations will not be taken into account in this study as their direct role in glucose regulation has not been fully established.

#### Beta cell mass

**Figure 2:**
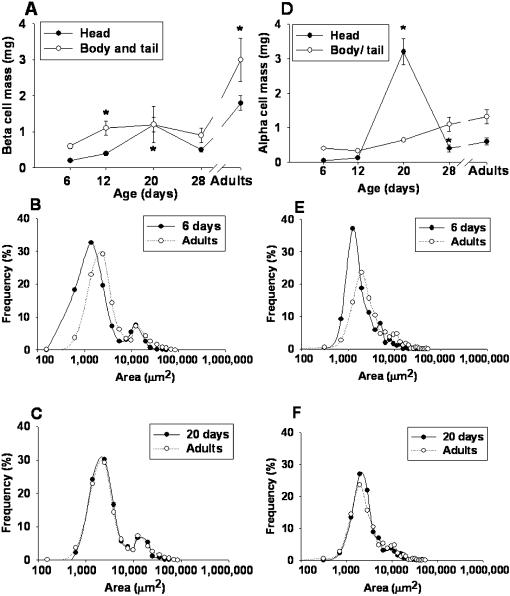
Structural changes of pancreatic beta and alpha cells during the first month of life. **(A)** The mass of beta cells in the head and tail of the gland. **(B)** Frequency distribution of beta cell aggregates at d6 and d90. **(C)** Comparison between d20 and d90. Data were pooled from 8 rats, n  =  9 216 beta cell aggregates measured. **(D)** Mass of alpha cells in the head and tail during the first month of life. **(E, F)** Frequency distribution of the size of alpha cell aggregates at d6, d20 and d90. **(E)** Comparisons between d6 and d90. **(F)** Comparisons between d20 and d90. Data were pooled from 4 rats, n  =  1 739 alpha cell aggregates measured, are expressed as mean ± SEM. Symbols denote statistically significant differences: (*) p < 0.05 with respect to previous age.

Beta cell mass increased from d6 to d20 in both, the head and body/tail. In the head, it peaked by d20 reaching values 4-fold higher than in d12 (Fig. 2A). Beta cell mass in the body/tail doubled between d6 and d12, and remained stable until d28 and then continued to increase until adulthood.

#### Size of beta cell aggregates

We observed a bimodal distribution of the size of beta cell aggregates at all ages studied. In all the distributions the predominant size was <10 000 µm^2^. However, a small fraction of cells formed larger aggregates ≥10 000 µm^2^. Compared to adult rats, at d6 the frequency of small aggregates was higher (Fig. 2B) while at d20 the distribution was similar (Fig. 2C).

#### Alpha cell mass

From d6 to adulthood, alpha cell mass increased in the body/tail of the gland. In contrast, the head showed a dramatic increase between d6 and d20 but at d28 a significant decline was observed, followed by a 1.5-fold increment to reach adulthood values (Fig. 2D).

#### Size of alpha cell aggregates

A bimodal distribution of the size of alpha cell aggregates was also observed at all ages studied; with a predominant size between 2 000 and 3 000 µm^2^. Less numerous larger aggregates, between 8 000–18 000 µm^2^, were also observed (Figs. 2E and F). At d6 most of the aggregates were small and at d20 the distributions were similar to that in adults.

#### Isolated beta or alpha cell clusters

We observed an important percentage of clusters of alpha cells or beta cells alone that do not exhibit the classical islet configuration. We quantified them (Table 2) and observed that on d20 beta cell clusters decreased in the head and increased in the body/tail region. Interestingly, alpha cell clusters in the head greatly increased from d6 to d20, followed by an important decrease in the adults. In the body/tail, alpha cell clusters steadily decreased from d6 to d20, where they reached adult values (nearly 25%).

**Table 2:**
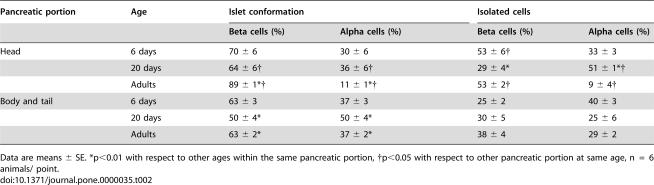
Changes in islet conformation and in isolated beta and alpha cells during the first month of life.

Pancreatic portion	Age	Islet conformation		Isolated cells	
		Beta cells (%)	Alpha cells (%)	Beta cells (%)	Alpha cells (%)
Head	6 days	70 ± 6	30 ± 6	53 ± 6†	33 ± 3
	20 days	64 ± 6†	36 ± 6†	29 ± 4*	51 ± 1*†
	Adults	89 ± 1*†	11 ± 1*†	53 ± 2†	9 ± 4†
Body and tail	6 days	63 ± 3	37 ± 3	25 ± 2	40 ± 3
	20 days	50 ± 4*	50 ± 4*	30 ± 5	25 ± 6
	Adults	63 ± 2*	37 ± 2*	38 ± 4	29 ± 2

Data are means ± SE. *p<0.01 with respect to other ages within the same pancreatic portion, †p<0.05 with respect to other pancreatic portion at same age, n  =  6 animals/ point.

#### Development of islets

**Figure 3:**
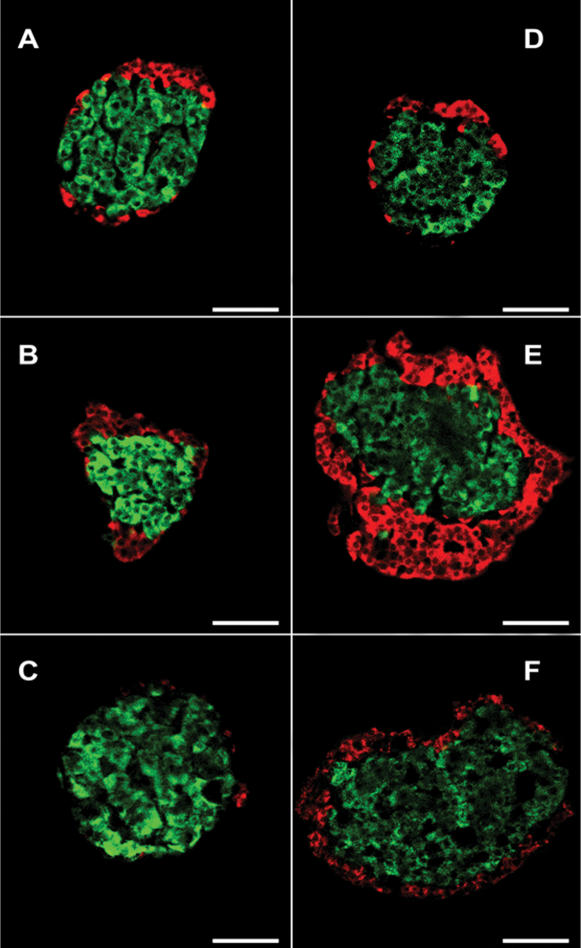
Islet composition changes in pancreatic head and tail at d6, 20 and adults. Photomicrographs illustrate the overall distribution of alpha (red) and beta (green) cells in representative islets of the head (A–C) and body/tail (D–F) at d6 (A and D), d20 (B and E) and adults (C and F). Scale bar  =  50 µm.

All the rest of beta and alpha cells grouped together to form islet-like structures (Table 2 and Figure 3). In the head, nearly 64% of beta cells were associated with alpha cells between d6 and d20. However, in adulthood, islets of the head were predominantly formed by beta cells, maybe associated to PP cells, as has been previously described [11]. In the body/tail of the gland, the percentage of alpha cells in contact with beta cells peaked around d20.

### Functional characteristics of beta cells during the first month

**Figure 4:**
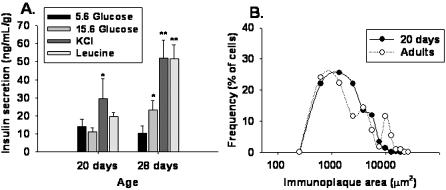
Insulin secretion and functional subpopulations from isolated beta cells at d20 and d28. (A) Bar graphs represent the release of insulin by isolated d20 and d28 beta cells in response to glucose, 40 mM KCl and 10 mM leucine, measured by ELISA; mean ± SEM, n  =  3 experiments by duplicate. Symbols denote statistically significant differences, (*) p<0.01 with respect to control, (**) p<0.001 with respect to control. **(B)** Frequency distribution of insulin immunoplaque areas of beta cells at d20 and d90 stimulated with 15.6 mM glucose, measured by a RHPA; n  =  4 experiments by duplicate.

**Figure 5:**
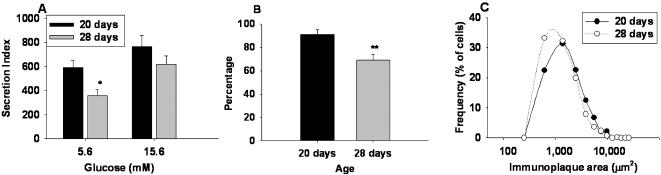
Insulin secretion at d20 and d28 explored by RHPA. (A) Bar graphs represent insulin secretion index at d20 and d28, in response to different glucose concentrations, mean ± SEM, n  =  4 experiments by duplicate, (*) p<0.05 with respect to 15.6 mM. **(B)** Percentage of insulin secreting beta-cells from the total population as identified by insulin immunocytochemistry after the RHPA; mean ± SEM, n  =  4 experiments by duplicate, **p<0.0001 with respect to d20. **(C)** Pancreatic beta cell subpopulations at d20 and d28 in response to basal, 5.6 mM glucose. Data pooled from 3 experiments by duplicate.

Insulin secreted to incubation media was measured by ELISA (see Methods). Isolated d20 beta cells secreted the same amount of insulin when exposed either to 5.6 (basal condition) or 15.6 mM glucose (Fig. 4 and Fig. 5). However, d20 cells in 5.6 mM glucose increased insulin secretion by 2.6-fold in response to a depolarizing concentration of KCl (Fig. 4A).

Isolated d28 beta cells responded by doubling insulin secretion to a high glucose concentration (15.6 mM), compared to basal glucose. Moreover, these cells increased by 5-fold insulin secretion in response to both, high KCl and leucine.

In order to analyze in detail insulin secretion by isolated beta cells, we used a reverse hemolytic plaque assay (RHPA), followed by a sequential insulin-immunocytochemistry to determine total beta cells (see Methods).

Analysis of immunoplaque areas that reflects insulin secretion by isolated beta cells at 15.6 mM glucose, revealed a multimodal frequency distribution that represents different functional subpopulations of beta cells [12], as shown in Fig. 4B (see Methods). As previously observed, adult beta cells could be grouped in two subpopulations, small and large immunoplaque-forming cells (SP and LP cells, respectively). The main subpopulation observed at d20 was formed by SP cells, with almost none LP cells. This observation is interesting because in the adult, LP cells are mainly recruited at higher glucose concentrations [12], [13].

A higher basal insulin secretion at 5.6 mM glucose (Figure 5A) was observed at d20, compared to cells from d28. This observation partially explains the inability of d20 cells to discriminate between different glucose concentrations. A similar tendency was observed using ELISA (Figure 4) however without statistical significance, which could be due to a lower sensitivity of insulin ELISA versus RHPA.

We observed by RHPA that 91% of immunocytochemistry-identified beta cells formed immunoplaques at d20, while this percentage decreased to 69% at d28 (Figure 5B). Finally, functional subpopulations at 5.6 mM glucose are shown in figure 5C, which reveals that at d20 there is a higher frequency of larger immunoplaque areas when compared with d28.

### Peripheral insulin sensitivity

**Figure 6:**
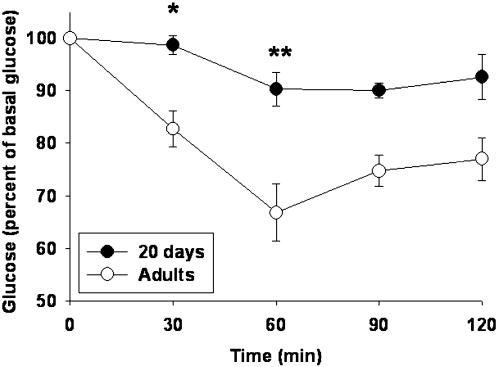
Insulin tolerance test (ITT) at d20 and adults. ITTs were performed in groups (n  =  6) of anesthetized 20d and adult rats. Insulin (0.2 U/kg) was administered iv via a femoral catheter and blood samples were withdrawn for glucose measurements at the times shown; (*) p<0.05 with respect to adults.

Peripheral insulin sensitivity was explored by an insulin tolerance test and results are shown in Fig. 6. In adults, a single insulin injection started immediately a decrease in blood glucose levels that reached a maximum decrease of 33% of basal glucose levels. In contrast at d20, compared to adults, the response was delayed by 30 minutes and it only reached a glucose nadir of 10%, which suggest a diminished peripheral sensitivity to insulin.

## Discussion

The weaning period represents a metabolic challenge because rats change from a constant lipid-rich milk diet, to an omnivorous diet, high in carbohydrates. In this developmental stage we detected an interesting physiological state of hyperinsulinemic hyperglycemia. This condition was associated with an increment in beta cell mass and a lack of response to high glucose concentrations. On the other hand, we also observed an increase in alpha cell mass and in the insulin counter-regulatory hormone glucagon. Functionaly the hyperinsulinemic period at d20 is correlated with a high basal insulin secretion by isolated beta cells.

To our knowledge this is the first description of this kind of phenomenon in normal development of the rat and indicates that weaning period is a critical window for pancreatic development. This is in accordance to other studies which have observed that high carbohydrate diets during lactation predispose organisms to obesity and type 2 diabetes in adulthood [7]–[10].

Previous studies have shown that pancreatic beta cells undergo an active proliferation and apoptosis during the first 20 days of life [6]. In this study, we present novel data that documents the reorganization of pancreatic islets, which could be partially explained by these previous results.

Our observations show that structural changes of beta and alpha cell mass and islet structure follow different patterns in the head and the body/tail of the gland. The reason for this phenomenon is unclear, but it may reflect differences in embryological development as the head and the body/tail derive from the ventral and dorsal buds, respectively, of the caudal foregut. The ventral bud relates anatomically to the mesoderm, whereas the dorsal bud is closely related with the notochord [2], indicating that each might be exposed to different growth factors during embryological development. In this regard, it would be important to study whether control of developmental patterns in each structure is independently determined.

We also observed that beta cell mass and the size of their aggregates increase during the first month of life and this expansion continues until adulthood, as it has been observed previously [14]. It is possible that this increase is due to neogenesis; as it has been shown that beta cell proliferation decreases after birth [6]. The peak of beta cell that we observed on d20 is slightly different from the one observed previously [6]. This difference could be due to variations in the animal models.

As it has been previously reported [11], [15], adult alpha cell mass was significantly larger in the body/tail than in the head of the gland. However, this relationship was inverted at d20 as a remarkable increase in alpha cell mass in the head was observed, compared with previous days. We consider that this event might be partly explained by an increase in alpha cells within the islets, but more importantly, by small groups of alpha cells dispersed throughout the pancreatic parenchyma, as shown in table 2. The drop in alpha cell mass after d20 could be due to apoptosis of this cell population or to cell-differentiation to other cell type, specifically pancreatic polypeptide (PP) cells, as has been previously proposed [16]. This possibility would explain why the massive increase in alpha cells is not accompanied by a higher plasma glucagon level.

It has been previously shown that diets with high contents of fat and protein promote an increase in alpha cell content in the rodent pancreas [17], [18]. It is then likely that changes in alpha cell population might be correlated to diet, as d20 coincides with a transition from a high-protein, high-fat diet during lactation, to a high-carbohydrate diet after weaning.

We observed that cell groups constituted exclusively by alpha or beta cells endure important changes during the first month of life. These populations might not be subjected to the classical paracrine islet regulation because they are not in close contact with other endocrine cell types. Although their functional role remains unknown, we noticed that changes in glucagon plasma levels resemble the pattern of structural reorganization of the isolated alpha cell groups in the body/tail region during the first month, which suggest that this population of isolated endocrine cells might be physiologically relevant.

Rat adult islets from the head contain almost no alpha cells, as has been classically described, while those in the body/tail are formed by beta cells surrounded by alpha cells [11]; paradoxically, during the first 20 days, islets in the head had a significant percentage of alpha cells that in the adult are probably substituted by PP cells.

Previous studies have shown that fetal and neonatal beta cells display immature functional features characterized by diminished sensitivity to glucose, scarce insulin secretion and the absence of a biphasic response to glucose [3]–[5]. In contrast, adult beta cells show a robust, biphasic insulin secretion [19], [20] and consistently respond to different glucose concentrations secreting different amounts of insulin. Therefore beta cells undergo a functional transition between birth and adult life.

The observed secretory activity in d20 and d28 suggests that this functional transition occurs at some point during this time frame. Furthermore, as d20 beta cells increase insulin secretion in response to KCl, we suggest that at this stage, cells have an inadequate insulin secretion coupling response to glucose, rather than a deficit in insulin gene transcription. Interesting candidates to study are glucokinase, the rate limiting step of glucose metabolism [21]–[23], glucose transporter GLUT2 whose expression has been shown to be diminished in the fetal pancreas [24] and ionic channels.

Previous studies have reported insulin sensitivity of beta cells previous to d20 [4], [25], [26] which suggest that the detected insensitivity is transient and specific for the weaning period. This phenomenon can be interpreted as an adaptation response of beta cells to the hyperglycemia documented between d6 and d20 (Table 1) and to the elevated basal insulin secretion observed at d20 when compared to d28 (Figure 5A). Supporting this possibility is the fact that sustained high blood glucose levels lead to decreased glucose sensitivity of beta cells in adult rats [27], [28]. Interestingly, this impairment is reversed when euglycemia is restored at d28 and in the adult [29].

Glucose insensitivity at d20 can also be due to the fact that functional subpopulations that secrete more insulin, LP cells, are not fully developed. An increase in these cells is observed from d20 to d28 coinciding with the gain of function of beta cells (Figure 4 B).

Our results are in agreement to previous observations [1], which support the concept that developing organs have critical periods with intense structural and functional reorganization. In the case of the pancreas, this circumstance may render it vulnerable to environmental stimuli, such as nutritional changes that could have harmful lifetime consequences.

Although no structural and functional changes have been described in the human pancreas, several studies suggest that, similar to what is observed in rodents; the nutritional status during the breastfeeding period in humans determines the development of glucose intolerance in adulthood [30]–[32] and in turn predispose the development of type 2 diabetes mellitus.

In conclusion, our study provides evidence of postnatal structural and functional modifications in the pancreas during the lactation period in rats. Changes in form and function are related in space and time, suggesting that this period could be a critical window in determining adult pancreatic physiology.

## Methods

All methods used in this study were approved by Animal Care Committee of the Instituto de Fisiologia Celular, Universidad Nacional Autonoma de Mexico. Animal care was performed according to the Guide for the Care and Use of Laboratory Animals (National Academy of Sciences, Washington D.C., 1996).

### Animals

The experiments were carried out in Wistar male rats at d6, d12, d20, d28 and d90 of postnatal life. Animals were born in our animal facility and raised under a 14 h light/10 h dark cycles with free access to food and water, they were weaned at d21. Rats were anesthetized with intraperitoneal sodium pentobarbital (40 mg/kg) before cardiac perfussion and pancreas dissection.

### Plasma concentration of glucose, insulin and glucagon

Blood samples for determination of glucose and hormones were obtained at the same time of day for all experiments (13.00 h) and under similar fasting conditions (4 h fasting). Blood was obtained through direct cardiac puncture; glucose levels were determined using a conventional monitoring system (MediSense Blood glucose Sensor Precision QID). Plasma insulin (ALPCO, Windham, NH) and glucagon (Wako Chemicals, Osaka, Japan) concentrations were determined by ELISA. Glucose and hormone concentrations were also expressed per gram of body weight to compensate for growth during the first month.

### Immunocytochemistry

Rats were perfused through cardiac puncture with Bouin's solution, the pancreas was dissected separating the pancreatic head (in contact with the duodenum) and the body/tail region (in contact with the spleen, the greater curvature of the stomach and the transverse colon). Samples were included in paraffin and serial longitudinal sections of 7 µm thick every 200 µm were obtained. Sections were incubated with guinea pig primary antibodies raised against insulin (1∶2 000, ICN, Aurora, OH) for 4 h at room temperature or mouse antibodies against glucagon (1∶6 000, Sigma, St. Louis, MO) at 4°C overnight. Primary antibodies were detected by using biotinylated goat anti-guinea pig or anti-mouse antibodies (1∶100) for 1 h at room temperature and revealed with the avidin-peroxidase complex (Vector, Burlingame, CA). For islet composition and isolated cells studies a second CY5-conjugated (excitation  =  650 nm, emission  =  670 nm) goat anti-mouse IgG antibody was added in the case of glucagon detection and a fluorescein isothiocyanate-conjugated (excitation  =  494 nm, emission  =  520 nm) goat anti-guinea pig IgG antibody in the case of insulin detection. Cells were observed under confocal microscopy.

### Beta and alpha cell morphometric analyses

To estimate the beta and alpha cell mass and the size of alpha or beta cell aggregates (defined as any group of alpha or beta cells within or outside islets), outlines (4X) of the entire section and of the clusters of stained cells within it were generated using a camera *lucida* attached to an Optiphot Nikon Microscope. These drawings were scanned, digitalized, and the area of the sections and of the alpha or beta cell clusters immunostained for glucagon or insulin was measured by a computer based imaging analysis system (Scion Image; Scion Corporation). The percentage of pancreatic area occupied by alpha or beta cells was estimated and cell mass was calculated by multiplying the percentage area of alpha or beta cells by the weight of the corresponding pancreas [33].

Islet conformation was studied by confocal analysis in consecutive slices stained for insulin and glucagon. The percentage of islets occupied by beta and alpha cells was calculated in 10 islets per pancreatic portion. Isolated beta and alpha cell groups were those immunostained cell groups not in contact with cells of the other endocrine type and their frequency was calculated in 10 fields per pancreatic portion.

### Pancreatic beta cell culture

Animals were anesthetized and the pancreas dissected. Samples were washed several times with Hank's balanced salt solution (HBSS; Sigma, St. Louis, MO) supplemented with 0.1% bovine serum albumin (BSA), incubated with collagenase IV (Worthingthon, Freehold, NJ) at 37°C and fractioned through a Ficoll (Sigma) gradient to isolate pancreatic islets. Clean islets were obtained, washed and mechanically dissociated in a calcium-free solution to obtain single cells, as described previously [34]. Single cells were cultured at 37°C, in RPMI-1640 (11.6 mM glucose) supplemented with 200 units/ml penicillin G, 200 mg/ml streptomycin, 0.5 mg/ml amphotericin B and 1% fetal bovine serum. This glucose concentration in the culture medium was used because it has proven to maintain their function and survival. *In vitro* insulin secretion experiments were carried out after 48 h of culture.

### Insulin secretion *in vitro*

Insulin concentration was measured in supernatants of media collected from 100 000 cells cultures of d20 and d28 beta cells exposed to a) 5.6 mM glucose, b) 5.6 mM glucose/40 mM KCl, c) 5.6 mM glucose/10 mM L-leucine, or d) 15.6 mM glucose for 1 h at 37°C. All of the cultures were equilibrated with Hanks' Solution (5.6 mM glucose) for 1 h at 37°C prior to the experiments. Insulin concentration was determined by the enzyme linked immunoabsorbent assay (ELISA) (ALPCO, Windham, NH). Results were expressed per gram of pancreatic weight to compensate for growth during the first month.

### Reverse hemolytic plaque assay for insulin (RHPA)

To identify insulin-secreting cells and to measure insulin secretion by single cells at d20 and d28, we used the reverse hemolytic plaque assay as previously described [13]. Briefly, 100 000 isolated islet cells were challenged for 1 h with 5.6 or 15.6 mM glucose in the presence of an insulin antiserum (1∶20) (Biogenesis, Sandown, NH), and further incubated for 30 min with guinea pig complement (Life Technologies, Grand Island, NY). Insulin released during the incubation time was revealed by the presence of hemolytic immunoplaques around secretory cells. The percentage of insulin secreting cells capable of forming immunoplaques was estimated and the overall secretory activity of beta cells under a given experimental condition was expressed as a secretion index. This index was calculated by multiplying the average immunoplaque area by the percentage of plaque-forming cells. To identify functional subpopulations of beta cells, a frequency histogram was constructed using the data on the number of cell-forming immunoplaques. With the insulin-antiserum that we are using, we classified them as follows: small plaque-forming cells (SP; immunoplaque ≤ 4 000 µm^2^), and large plaque-forming cells (LP; immunoplaque >4 000 µm^2^). In order to quantify the number of beta and alpha cells in the experiments we made immunocytochemistry for insulin and immunofluorescence for glucagon, on the insulin plaque-forming cells [35]. In these experiments the RHPA was done as described on coverslips previously marked with letters, to make the identification of single cells possible and the correlation with the immunostaining. Then cells were fixed with paraformaldehyde and developed as described.

### Insulin tolerance test (ITT)

Rats were anesthetized with ether and the right femoral vein catheterized. Blood samples were withdrawn at 1 min before and 30, 60, 90 and 120 min after an injection of lispro-insulin (0.2 U/kg; Human Lispro insulin, Lilly, Fegerheim, France) [36]. Blood samples were taken from the tail for glucose measurements.

### Statistical analysis

Data are reported as means ± SE. Statistical significance was assessed with one-way ANOVA, followed by Fisher's multiple range test or Students t-test as specified, using the program Statview 4.57 (Abacus Concepts, Cary, NC).
